# COVID-19 and patients on renal replacement therapy: Perspective from the State of Qatar

**DOI:** 10.5339/qmj.2024.1

**Published:** 2024-05-09

**Authors:** Abdullah Hamad, Mohamad M. Alkadi, Hassan Al-Malki

**Affiliations:** 1 Division of Nephrology, Department of Medicine, Hamad General Hospital, Doha, Qatar Email: MAlkadi@hamad.qa

Coronavirus disease 2019 (COVID-19) is an infectious disease caused by a single-stranded RNA virus called severe acute respiratory syndrome coronavirus 2 (SARS-CoV-2) that typically causes respiratory damage and can lead to multisystem disorders, especially in immunocompromised patients. The coronavirus disease 2019 (COVID-19) infection was first reported in Wuhan, China, in December 2019, and by March 2020, the World Health Organization (WHO) declared COVID-19 a pandemic given the global widespread.

COVID-19 caused high morbidity and mortality among patients on renal replacement therapy, such as dialysis and kidney transplant patients. The reported mortality rates ranged between 20- 40% in early reports from multiple geographic areas worldwide.^1-3^ A cumulative report by Jager et al. from the ERA-EDTA registry showed COVID-19-attributable mortality rates of 20% of 3285 dialysis patients and 19.9% of 1013 kidney transplant recipients.^4^ However, the mortality rates among Qatar’s dialysis and kidney transplant patients infected with SARS-CoV-2 in early COVID-19 waves were 15% and 2.3%, respectively. We did not find a significant difference in mortality rates between hemodialysis (HD) and peritoneal dialysis patients. Twenty-five percent of dialysis patients and 21% of kidney transplant recipients with COVID-19 required intensive care unit (ICU) admission.^5,6^

As many COVID-19 patients required admission to intensive care units (ICU) early in the pandemic, ICU beds were increased in Qatar by 300%. Therefore, nephrology services were restructured to overcome the significant increase in ICU admissions and the demand for renal replacement therapies. Special training was provided to ICU nurses by the dialysis staff on performing continuous renal replacement therapy (CRRT), and we succeeded in expanding the capacity of CRRT by 400%. Bedside acute intermittent HD and ambulatory HD capacities were also increased by 100% and 30%, respectively. In addition, ambulatory HD was made available in isolation facilities through portable dialysis machines.^7^

Afterward, SARS-CoV-2 underwent several rapid mutations due to its large genome RNA and low-fidelity RNA polymerase. Four variants of concern (VOC) were identified between January and September 2021 (alfa, beta, gamma, and delta), which were more transmissible and less immunogenic. In November 2021, WHO announced a new COVID-19 VOC named "Omicron," characterized by high infectivity but less virulence.^8^ Between December 2021 and January 2022, 20% of HD patients and 40% of dialysis staff were diagnosed with COVID-19. Our nephrology service took several steps to cope with the massive spread of COVID-19 among HD patients and staff. A national multidisciplinary nephrology task force was assembled and had to meet regularly for updates on disease spread and proper resource utilization. Some of the temporary steps taken were allocating one facility to handle all Omicron-infected patients, increasing the ratio of nurses to patients, extending working hours from 8 to 12 hours, reducing dialysis sessions from 4 to 3 hours (if a patient’s condition allows), adding a fourth dialysis shift (late evening), implementing new pathways for management of Omicron cases with proper education for staff.^9^ All these measures led to excellent control of the Omicron wave in Qatar. HD services gradually started to go back to normal in February 2022. Compared to the pre-Omicron era, we found that Omicron wave was associated with lower mortality (2.4% versus 14.5%), ICU admission (2.3% vs. 25.2%), and need for mechanical ventilation (1.7% vs. 16%). Thus, Omicron had a higher transmission rate but less morbidity and mortality compared to previous waves of COVID-19 infection.^10^

In late 2020, COVID-19 mRNA vaccines [BioNTech-162b2 (Pfizer) and the mRNA-1273 (Moderna)] obtained authorization after they showed ≥ 95% efficacy in preventing COVID-19 infection and severe complications in the general population.^11,12^ However, dialysis patients were excluded from earlier studies for safety considerations. Later, studies showed vaccine effectiveness in dialysis patients.^13,14^ In Qatar, the national COVID-19 vaccination campaign started in December 2020, and mRNA vaccines were the only approved and available vaccines.^15^ Immunocompromised patients, including dialysis and kidney transplant recipients, were given the highest priority in receiving COVID-19 vaccines. We reported our experience in Qatar, which showed a high vaccination rate in the HD population (91%). The effectiveness of mRNA vaccines against SARS-CoV-2 infection was estimated at 94.7% ≥ 14 days after the second dose.^16^ On the other hand, vaccine effectiveness in kidney transplant recipients in Qatar against SARS-CoV-2 was estimated at 46.6% (95% CI: 0.0-73.7%) ≥ 14 days after the second dose and vaccine effectiveness against any severe, critical, or fatal COVID-19 disease was estimated at 72.3%.^17^

In conclusion, Qatar’s experience underscores the importance of proactive planning, rapid response capabilities, and prioritizing vaccination in protecting patients on renal replacement therapy during public health crises like the COVID-19 pandemic. These lessons can guide future pandemic preparedness efforts and enhance healthcare systems worldwide.
